# Supplementary material for: Comparative Proteomic Identification of Ram Sperm before and after In Vitro Capacitation
Source: Animals (Basel). 2024 Aug 15;14(16):2363. doi: 10.3390/ani14162363 (PMC11350773; doi:10.3390/ani14162363)
Supplement: Supplementary file 1 [file animals-14-02363-s001.zip › Figure_S6.pdf]

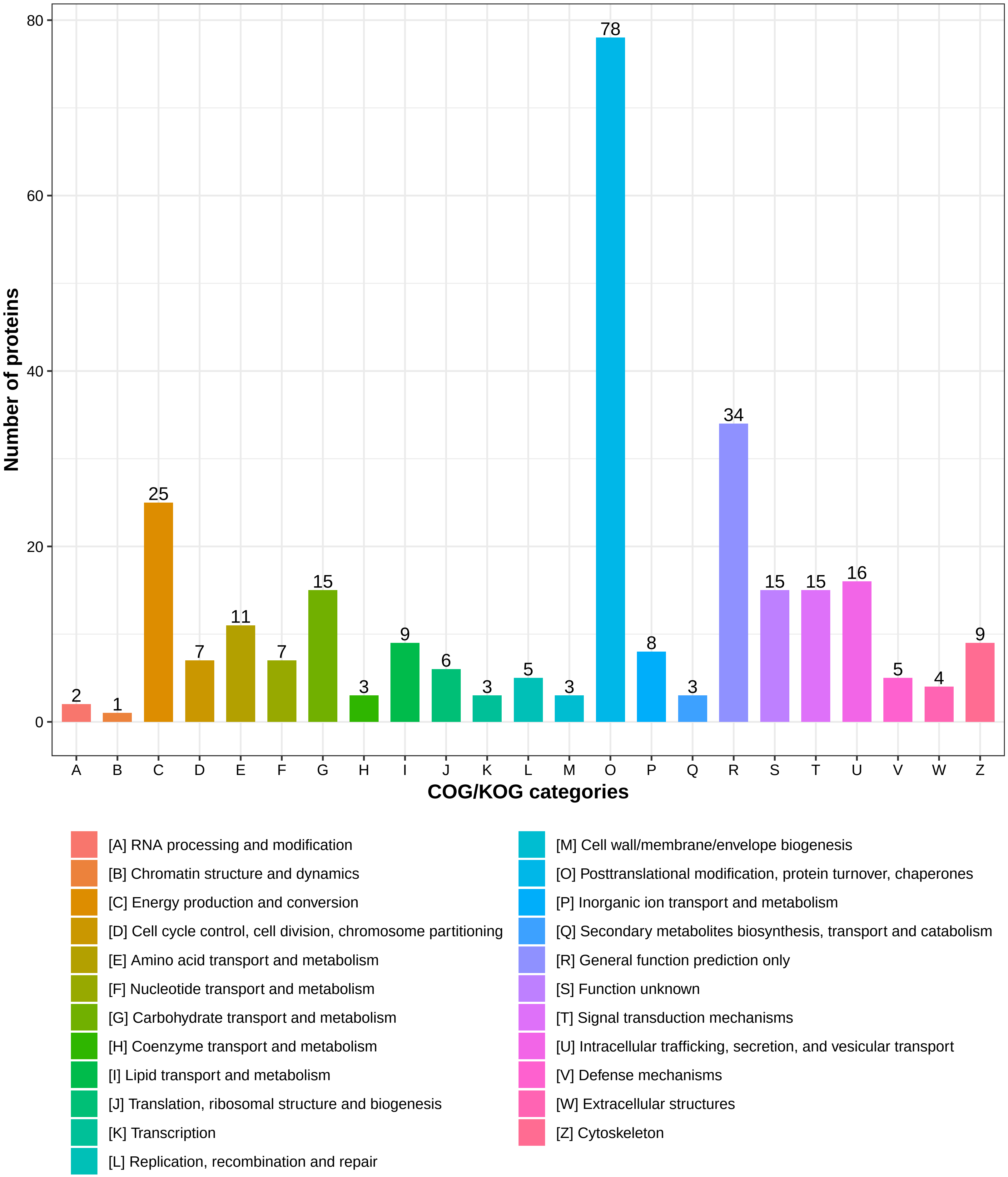

Number of proteins
80
60
40
20
0
78
34
25
15
16
11
9
7
8
6
5
4
3
2
1
A B C D E F G H I J K L M O P Q R S T U V W Z
COG/KOG categories
[A] RNA processing and modification
[B] Chromatin structure and dynamics
[C] Energy production and conversion
[D] Cell cycle control, cell division, chromosome partitioning
[E] Amino acid transport and metabolism
[F] Nucleotide transport and metabolism
[G] Carbohydrate transport and metabolism
[H] Coenzyme transport and metabolism
[I] Lipid transport and metabolism
[J] Translation, ribosomal structure and biogenesis
[K] Transcription
[L] Replication, recombination and repair
[M] Cell wall/membrane/envelope biogenesis
[O] Posttranslational modification, protein turnover, chaperones
[P] Inorganic ion transport and metabolism
[Q] Secondary metabolites biosynthesis, transport and catabolism
[R] General function prediction only
[S] Function unknown
[T] Signal transduction mechanisms
[U] Intracellular trafficking, secretion, and vesicular transport
[V] Defense mechanisms
[W] Extracellular structures
[Z] Cytoskeleton
