# Supplementary figures and images for: Comparative Proteomic Identification of Ram Sperm before and after In Vitro Capacitation
Source: Animals (Basel). 2024 Aug 15;14(16):2363. doi: 10.3390/ani14162363 (PMC11350773; doi:10.3390/ani14162363)

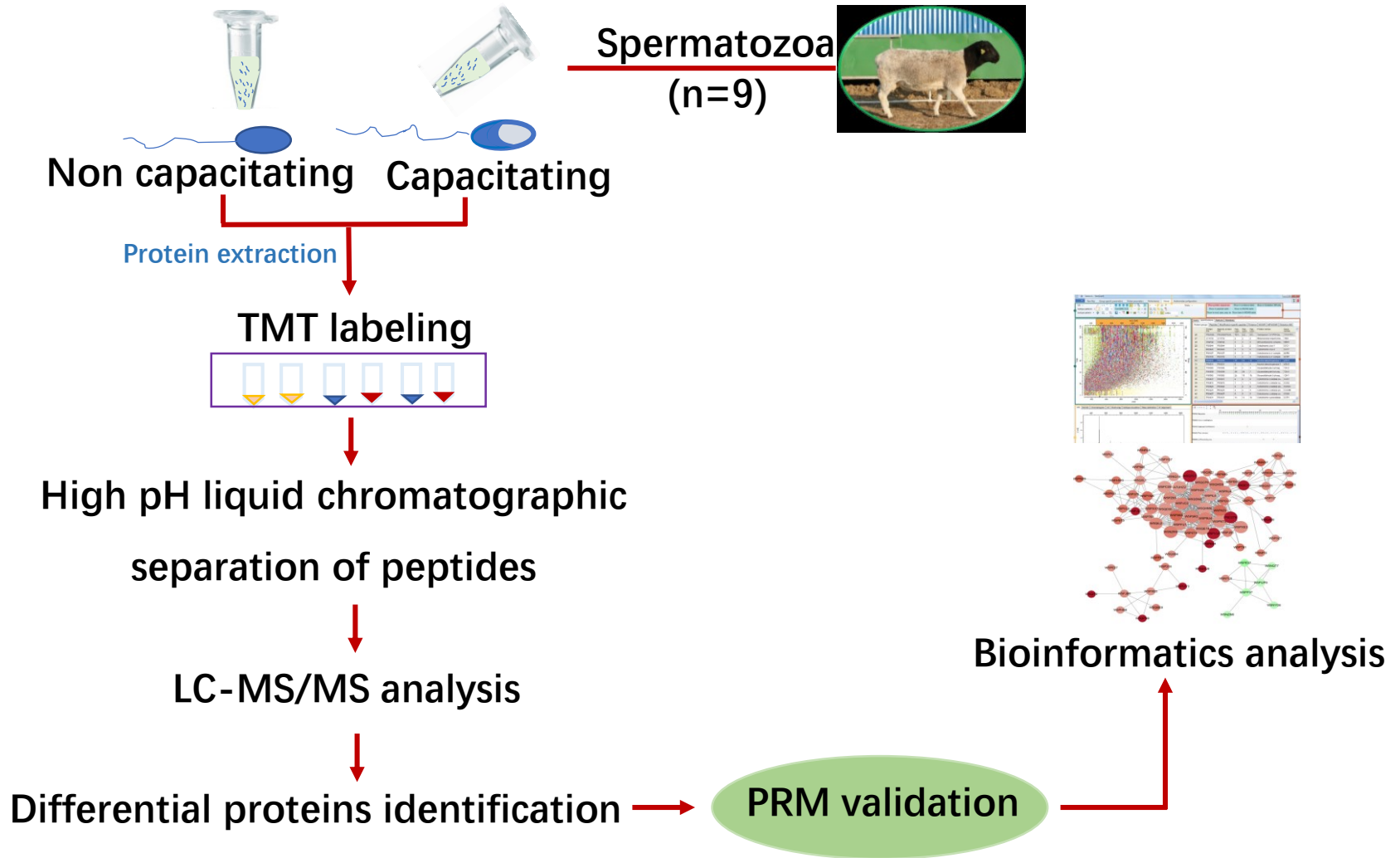

Supplement: Supplementary file 1 [file animals-14-02363-s001.zip › Figure_S1.pdf]

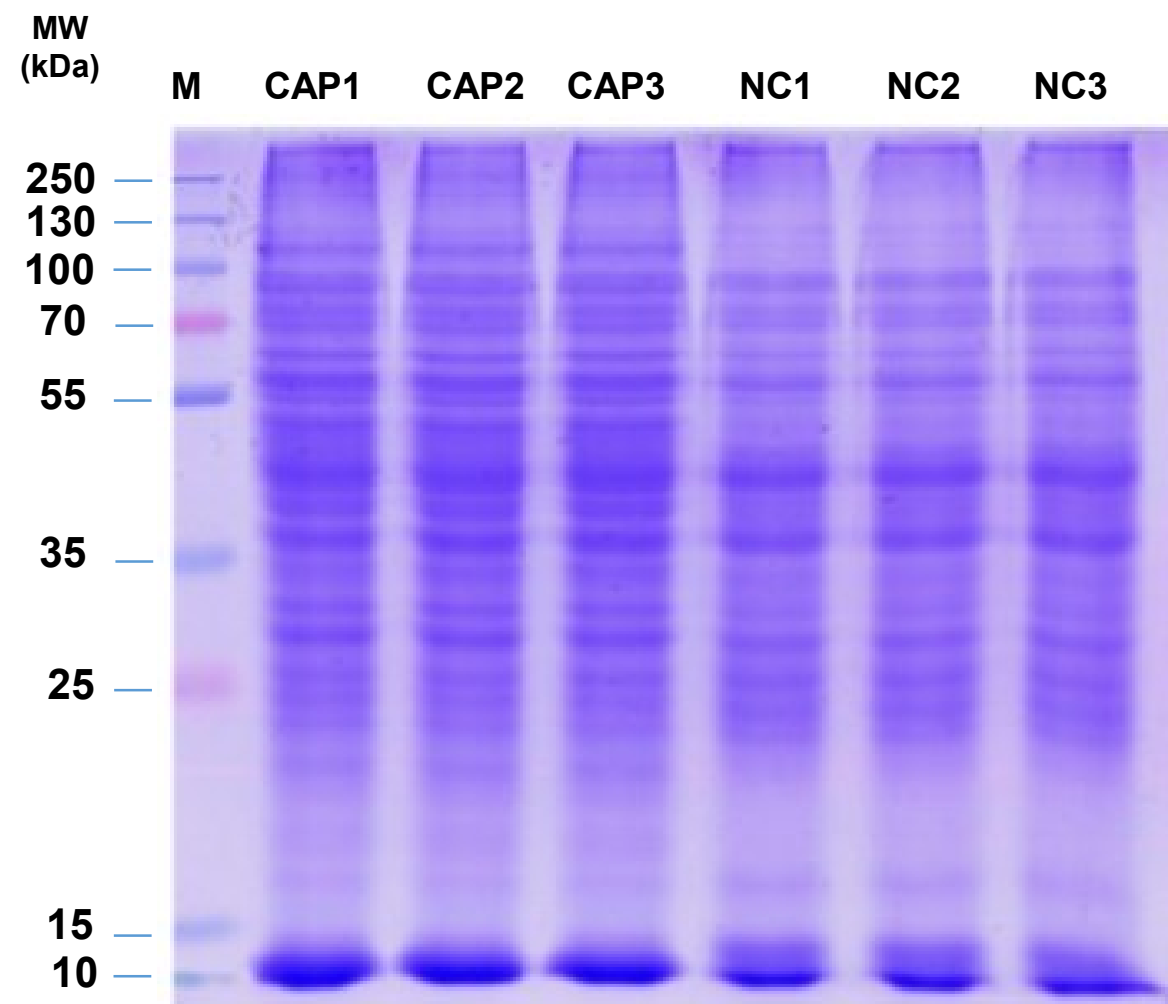

Supplement: Supplementary file 1 [file animals-14-02363-s001.zip › Figure_S2.pdf]

(A) NC

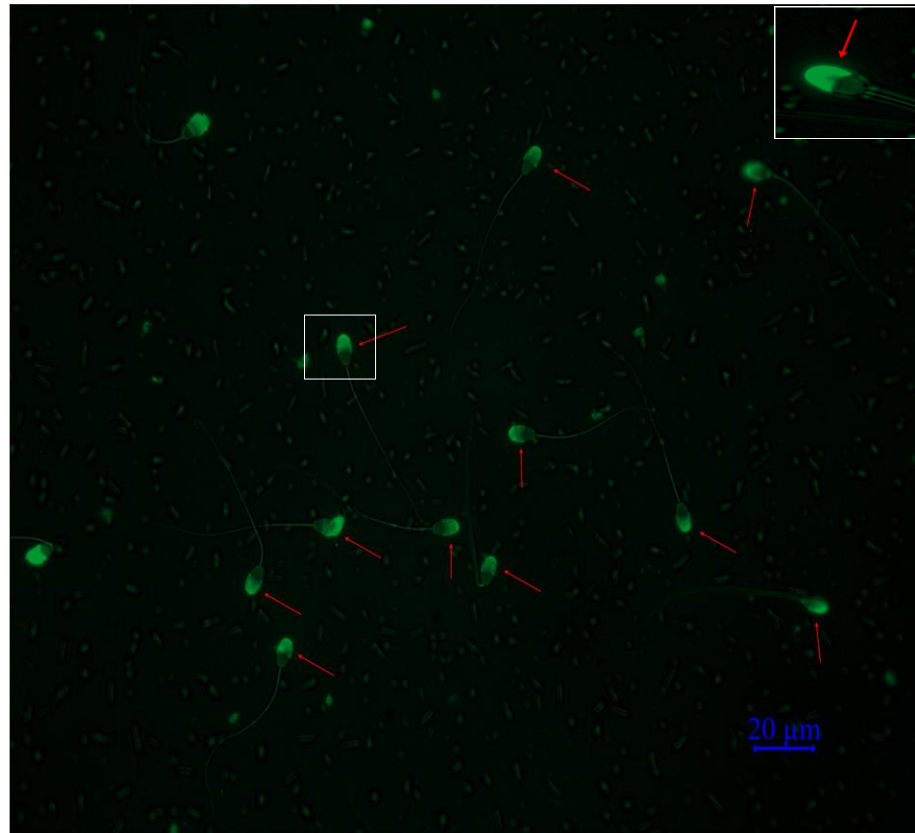

(B) CAP

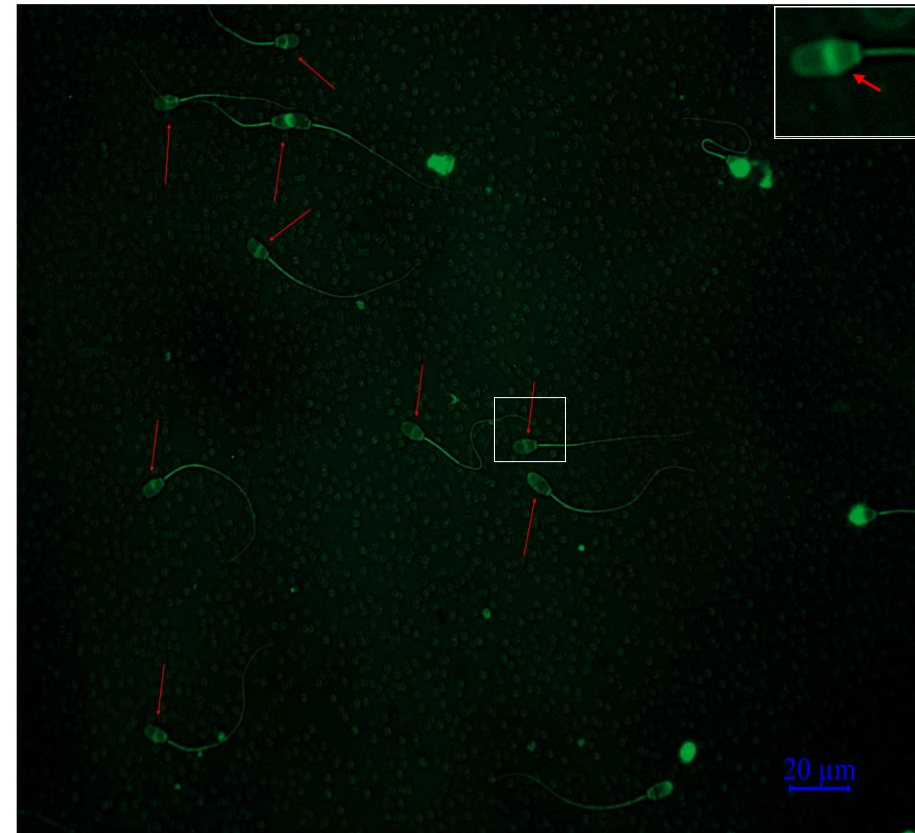

Supplement: Supplementary file 1 [file animals-14-02363-s001.zip › Figure_S3.pdf]

**A**

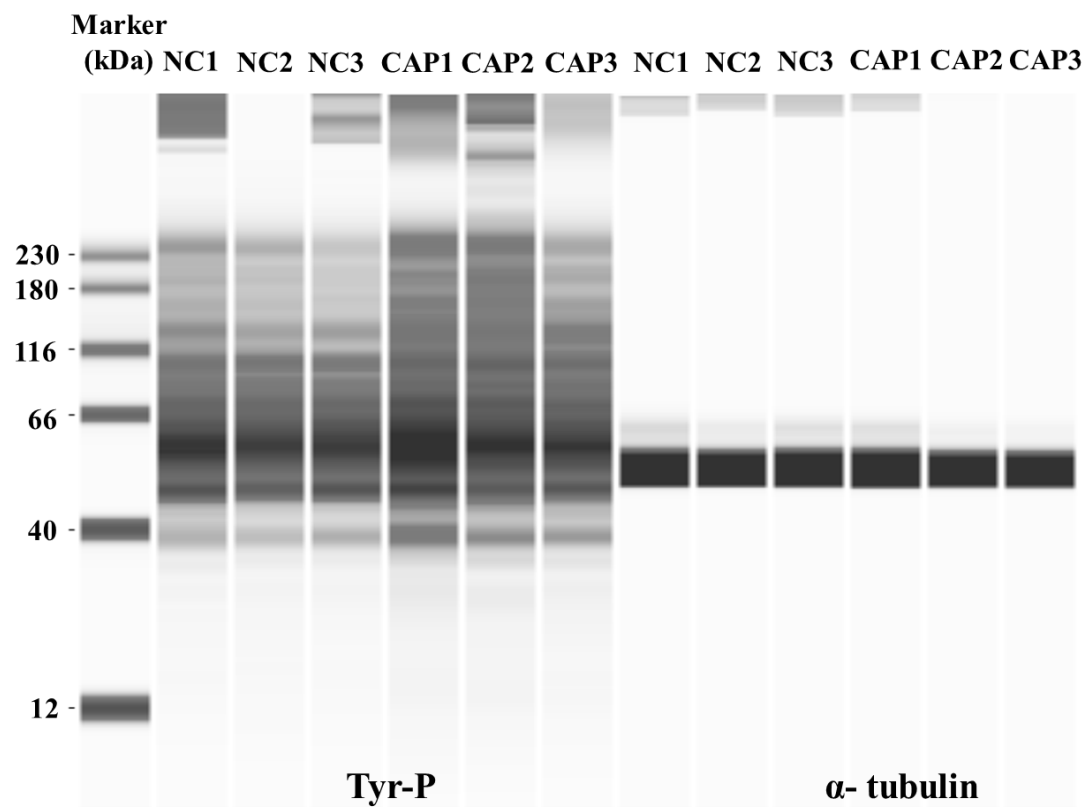

**B**

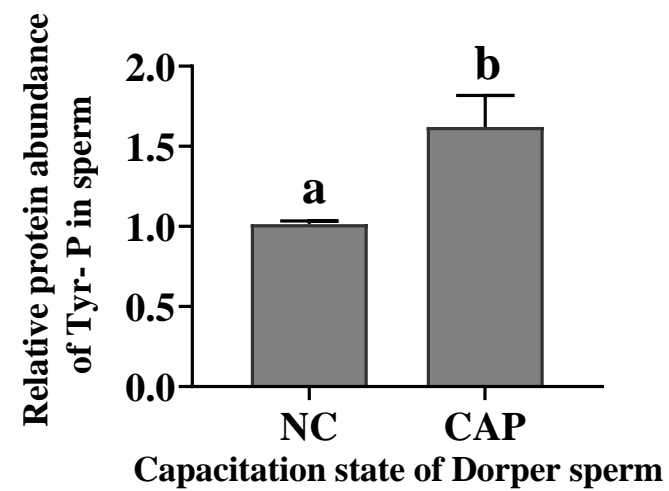

Supplement: Supplementary file 1 [file animals-14-02363-s001.zip › Figure_S4.pdf]

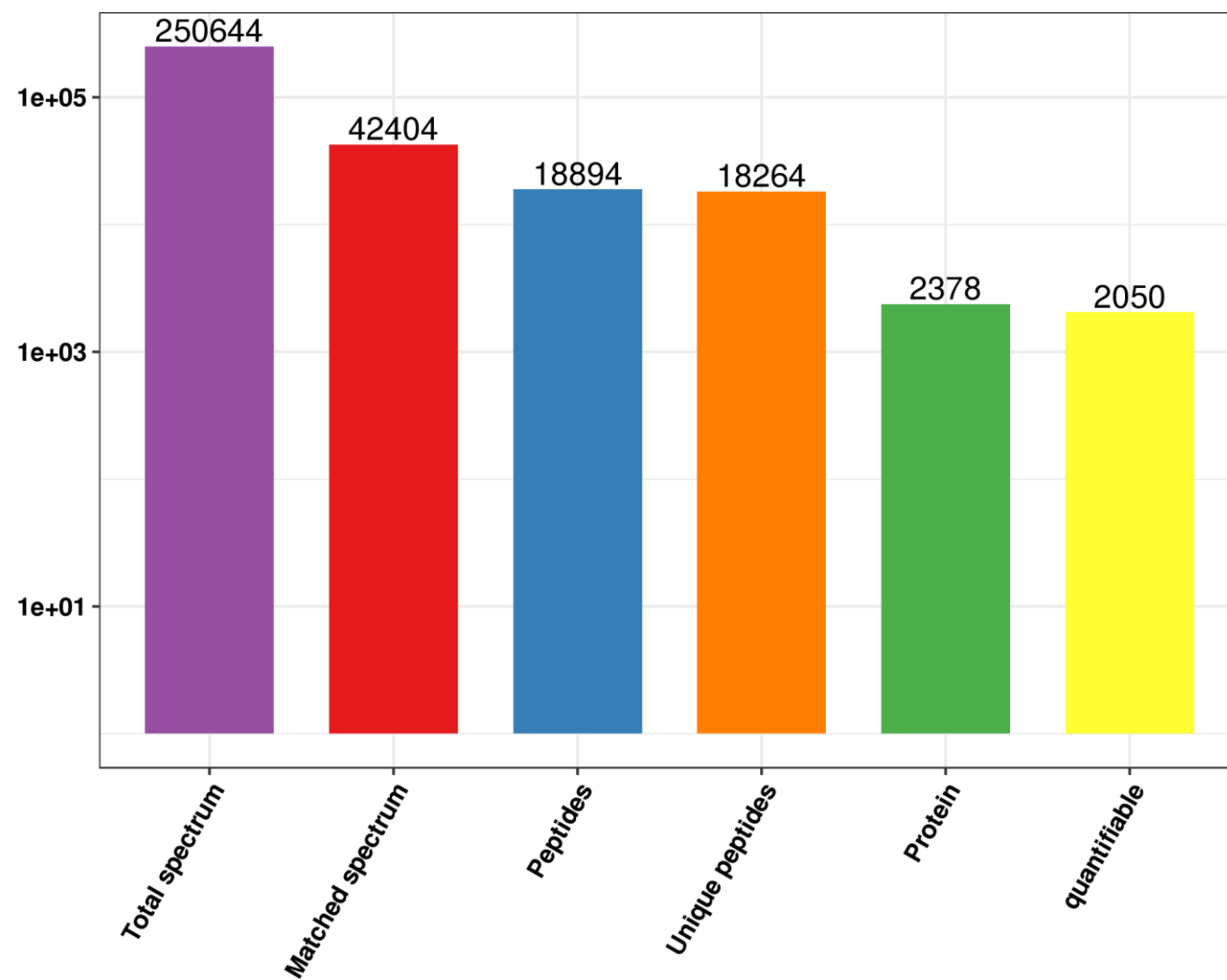

Supplement: Supplementary file 1 [file animals-14-02363-s001.zip › Figure_S5.pdf]
